# Mentalizing difficulties are transdiagnostic and explain links between mental health and neurodevelopmental symptoms and social adjustment in school‐aged children

**DOI:** 10.1002/jcv2.70034

**Published:** 2025-08-01

**Authors:** Rory T. Devine, Imogen Byrne, Venelin Kovatchev

**Affiliations:** ^1^ Centre for Developmental Science School of Psychology University of Birmingham Edgbaston UK; ^2^ School of Computer Science University of Birmingham Edgbaston UK

**Keywords:** executive function, neurodevelopmental conditions, psychopathology, social behavior, theory of mind

## Abstract

**Background:**

Mental health and neurodevelopmental conditions are a leading cause of disability in school‐aged children and are associated with adverse social outcomes. The aim of the current study was to investigate the extent to which mentalizing (also called ‘theory of mind’), the ability to reason about others' mental states is associated with specific mental health and neurodevelopmental symptoms or with a general vulnerability to psychopathology (the ‘P‐ Factor’) and whether mentalizing might explain why children with diverse mental health and neurodevelopmental conditions experience adverse social outcomes.

**Methods:**

In a pre‐registered study using a transdiagnostic dimensional approach, we collected direct assessments of mentalizing, multi‐informant measures of social adjustment at school, and teachers' ratings of mental health and neurodevelopmental symptoms from a diverse community sample of 1020 8‐ to 13‐year‐old children (54.5% girls).

**Results:**

High scores on the P‐Factor (a general vulnerability to mental health and neurodevelopmental conditions) were negatively associated with children's mentalizing, β = −0.154, 95%CI [−0.219, −0.089]. Once the P‐Factor was considered, there were no unique associations between P‐free symptom factors (i.e., internalizing, externalizing, attention deficit/hyperactivity traits, or autism traits) and mentalizing or social adjustment. Mentalizing partly explained the association between P‐Factor scores and poor social adjustment at school, β = −0.072, 95%CI [−0.116, −0.028].

**Conclusions:**

The results indicate that theory‐of‐mind difficulties are transdiagnostic and underscore the need for longitudinal work examining whether mentalizing explains links between mental health, neurodiversity and social adjustment.

## Key points

**What's known**

- Growing evidence suggests that children's difficulties reasoning about others' thoughts, feelings and desires (‘mentalizing’) cut across many mental health and neurodevelopmental conditions but studies have typically focused on single conditions.

**What's new**

- This study adopted a dimensional approach to investigate the links between mentalizing, mental health and neurodevelopmental symptoms, and social adjustment in a large community sample of children and adolescents.
- Mentalizing difficulties were transdiagnostic and partly accounted for the link between mental health and neurodevelopmental symptoms and social adjustment.

**What's relevant**

- The results challenge condition‐specific accounts of mentalizing difficulties and underscore the need for longitudinal work examining whether mentalizing explains links between mental health and neurodevelopmental conditions and social adjustment.

## Graphical abstract

Growing evidence suggests that difficulties reasoning about others' thoughts, feelings and desires (called ‘mentalizing’ or ‘theory of mind’) cut across many mental health and neurodevelopmental conditions but studies have yet to test this claim directly. This study adopted a dimensional approach to investigate the links between mentalizing, mental health and neurodevelopmental symptoms, and social adjustment in a large community sample of children and adolescents. Mentalizing difficulties were transdiagnostic and partly accounted for the link between mental health and neurodevelopmental symptoms and social adjustment. The results challenge condition‐specific accounts of mentalizing difficulties and underscore the need for longitudinal work examining whether mentalizing explains links between mental health and neurodevelopmental conditions and social adjustment.

## INTRODUCTION

Approximately 1 in 8 children and young people (13.4%) between the ages of 6 and 18 years have mental health or neurodevelopmental conditions (Polanczyk et al., 2015) and for 1 in 3 of these children and young people, their condition first emerged before the age of 14 years (Solmi et al., 2022). Mental health and neurodevelopmental conditions are a leading cause of disability in school‐aged children and are associated with friendlessness, peer rejection, and difficulties with everyday social interactions (Szekely et al., 2016). Yet, the processes underpinning the links between mental health difficulties, neurodevelopmental conditions and social adjustment in school‐aged children remain poorly understood. The aim of the current study was to investigate the extent to which individual differences in understanding others' mental states, called ‘theory of mind’ (ToM) or ‘mentalizing’, are associated with mental health and neurodevelopmental symptoms and the degree to which mentalizing sheds light on why children with diverse mental health and neurodevelopmental conditions experience adverse social outcomes.

### Mentalizing and children's mental health

Prominent accounts of autism proposed that differences in communication and social interaction experienced by autistic people were attributable to variations in mentalizing (Baron‐Cohen, 1995). Supporting this view, meta‐analyses have shown that autistic children and adolescents perform worse than their non‐autistic peers across a range of mentalizing tasks (Wilson, 2021). While divergent mentalizing was initially seen as specific to autism (Baron‐Cohen, 1991), subsequent work showing differences in mentalizing in children and adolescents with externalizing problems, internalizing problems, and other neurodevelopmental conditions suggests that difficulties reading others' minds might be transdiagnostic (Cotter et al., 2018).

The past decade has seen a growth of interest in transdiagnostic dimensional approaches to mental health and neurodevelopmental conditions (Apperly et al., 2024; Caspi et al., 2024). Unlike case‐control and cross‐syndrome studies comparing tightly defined groups with specific conditions, transdiagnostic dimensional studies leverage normal variation in symptoms in large community samples to identify spectra (i.e., ‘latent factors’) of mental health and neurodiversity that account for the co‐occurrence of symptoms (Astle et al., 2022; Gillan & Seow, 2020). This approach has been motivated by key criticisms of ‘standard’ categorical approaches, which fail to account for (1) the dimensional nature of symptoms, (2) the heterogeneity within traditional conditions, and (3) the widespread co‐occurrence of conditions (Astle et al., 2022; Gillan & Seow, 2020). Dimensional approaches to mental health initially sought to identify factors that explained commonly co‐occurring difficulties, such as ‘internalizing’ and ‘externalizing’, later called ‘first‐order’ symptom factors (Caspi et al., 2024). Building on work by Caspi et al. (2014), studies using bifactor modeling, where symptoms are modeled in terms of both a general factor and a symptom‐specific factor, have shown that diverse mental health and neurodevelopmental symptoms in children and adolescents load onto both a general dimension of psychopathology, called the ‘P‐factor’, and orthogonal (uncorrelated) ‘P‐Free’ symptom factors (e.g., internalizing, externalizing, inattention/hyperactivity, autism) (Bloemen et al., 2018).

Unlike first‐order symptom factors, orthogonal ‘P‐Free’ symptom factors capture variance that is not shared with other symptoms that load onto the P‐factor (Caspi et al., 2024). The P‐factor model therefore makes it possible to examine the degree to which phenotypes, like mentalizing, are associated with specific symptom dimensions that do not overlap with other dimensions (e.g., autism traits) or transcend mental health and neurodevelopmental conditions as evidenced by links with the P‐factor (Caspi et al., 2014; Caspi & Moffitt, 2018). Dimensional approaches have informed theorizing about relations between mentalizing and externalizing, internalizing, and psychoticism spectra (Sharp & Hernandez, 2021). To our knowledge, research comparing associations between children's mentalizing and specific symptom dimensions or the P‐factor has yet to be reported. Our first objective was to determine the specificity of the relations between mentalizing, mental health and neurodevelopmental symptoms using a dimensional approach in a large community sample of school‐aged children.

Mentalizing test performance in childhood is correlated with executive function (EF) (Devine & Hughes, 2014) and verbal ability (Milligan et al., 2007). Similarly, emotion recognition and mentalizing are viewed as a distinct but related socio‐cognitive skills (Oakley et al., 2016). Given that a range of mental health and neurodevelopmental conditions are associated with EF (Bloemen et al., 2018), verbal ability (Caspi et al., 2014) and emotion recognition (Zoupou et al., 2024), we included direct assessments of these skills to establish the extent to which mental health and neurodevelopmental symptoms are uniquely related to mentalizing.

### Mental health, mentalizing and social adjustment

Childhood mental health and neurodevelopmental conditions are linked with poor social adjustment including difficulties with peer relationships and social interaction (Xerxa et al., 2021). The social account of mentalizing proposes that variation in mentalizing matters for children's social lives (Hughes & Devine, 2015). Compared with their peers, children who are skilled at mentalizing are rated as more socially competent by teachers (Devine et al., 2016) and are more popular (Slaughter et al., 2015). Studies have yet to investigate the role of mentalizing in the relation between mental health and neurodevelopmental symptoms and social adjustment. Our second objective was to investigate the extent to which mentalizing accounted for links between mental health and neurodevelopmental symptoms and children's social adjustment. Given documented associations between EF and social adjustment (Lecce et al., 2020) and between emotion recognition and social adjustment (Trentacosta & Fine, 2010), we sought to rule out these confounding variables to establish unique associations between mentalizing and social adjustment (Devine et al., 2016).

### Research objectives and hypotheses

The current pre‐registered study adopted a transdiagnostic dimensional approach to investigate the links between mental health and neurodevelopmental symptoms and mentalizing in a large cross‐sectional community sample of school‐aged children. The first objective was to investigate the specificity of the relations between mentalizing, mental health and neurodevelopmental symptoms while controlling for EF, verbal ability, and emotion recognition. If mentalizing difficulties crosscut traditional conditions (i.e., the transdiagnostic account), then P‐factor scores will be more strongly associated with mentalizing than P‐free symptom dimensions. If mentalizing is linked only with P‐free symptom dimensions (i.e., ‘the specificity account’), then these dimensions will be more strongly associated with mentalizing than the P‐factor. The second objective of the current study was to examine the extent to which mentalizing accounts for links between mental health and neurodevelopmental symptoms and social adjustment. If difficulties with mentalizing arising from mental health problems and neurodevelopmental symptoms impede social adjustment (i.e., the social account), then mentalizing will partly account for the association between these symptoms and social adjustment.

## MATERIALS AND METHODS

### Participants

We recruited 1100 English‐speaking children between the ages of 8 and 13 years from 37 classrooms in state‐funded primary and secondary schools in the UK as part of a pre‐registered study of children's mentalizing, mental health, and social adjustment (https://osf.io/rxyfh) (Supporting Information S1: Appendix 1 for Power Analysis). Of the 1100 children in participating classrooms, 31 children were excluded because their caregivers did not provide consent for their participation and/or the children were unable to participate in the study unaided by a classroom assistant. A further 49 children declined to participate in the study. 1020 children participated in the study (Table 1 for Participant Characteristics).

**TABLE 1:** Participant characteristics and descriptive statistics for main study variables.

	N	M/%	SD	Range
Age (Years)	1020	10.36	1.27	8.27–13.27
Gender	1020			
Male		44.4%		
Female		54.5%		
Prefer not to say		1.1%		
Eligible for free school meals	770	23.2%		
Additional languages	772	28.9%		
Ethnicity	730			
White		51.5%		
Asian or Asian British		31.5%		
Black, Black British, Caribbean or African		8.1%		
Mixed or multiple ethnicity		6%		
Other ethnic group		2.9%		
Special educational needs	768	18.2%		
CBCL TRF (summed)				
Anxious‐depressed	782	3.24	4.23	0–24
Withdrawn‐depressed	782	1.12	1.92	0–13
Social dependence	782	1.26	2.37	0–16
Thought problems	782	0.75	1.88	0–14
Aggression	782	2.71	5.97	0–35
Rule breaking	782	1.14	2.21	0–14
Inattentiveness	782	4.38	6.44	0–28
Impulsivity‐hyperactivity	782	2.93	5.06	0–24
CAST (summed)				
Restricted, repetitive behavior	782	0.94	1.92	0–15
Communication skills	782	0.54	1.12	0–6
Reciprocal social interactions	782	1.41	1.60	0–6
Mentalizing				
Silent film task (summed)	977	6.09	2.56	0–12
Strange stories task (summed)	980	5.65	1.95	0–10
Triangles task (summed)	854	12.09	3.81	0–20
Social adjustment				
Peer social preference (z)	910	0.02	1.56	−5.76 to 3.65
Sociability‐leadership (z)	947	0.02	0.50	−0.79 to 2.25
Social maturity (mean)	773	4.17	1.24	1–7
Covariates				
Mill hill vocabulary (summed)	871	12.75	3.78	0–20
Emotion recognition (summed)	886	21.08	4.51	0–29
Executive function (z)	927	−0.00	0.35	−1.09 to 1.14

*Note*: Details about the scoring of the CBCL TRF and CAST are reported in the Supporting Information S1: Appendix. Details about the scoring of the executive function tasks can also be found in the Supporting Information S1: Appendix.

### Procedure

Children participated in two whole‐class sessions, approximately 1 week apart as part of an observational study. Researcher‐led testing sessions lasted between 60 and 90 min and included a fixed‐order battery of tasks. Children faced a large screen and completed all tasks on a personal computer. Teachers completed a questionnaire about each child.

### Ethics statement

The University of Birmingham Science, Technology, Engineering and Mathematics Ethical Review Committee provided ethical approval (ERN 09‐048AP10) on 16th August 2019. An opt‐out informed caregiver consent procedure was used whereby caregivers were given at least 2 weeks to contact the classroom teacher or study team to withdraw consent for their child to participate.

### Measures

#### Mental health and neurodevelopmental diversity

Teachers completed the *Child Behavior Checklist Teacher Report Form (CBCL TRF)* for children aged 6–18 years (Achenbach & Rescorla, 2013). There were 8 subscales: anxious‐depressed, withdrawn, aggressive behavior, rule‐breaking behavior, inattention, hyperactivity‐impulsivity, social dependence, and thought problems. We omitted four low frequency items that were not developmentally appropriate (i.e., talks of suicide, thinks of sex, uses tobacco, and uses drugs) and we did not administer the somatic complaints subscale. Teachers completed the 20‐item *Childhood Autism Syndrome Test* (CAST) (Ronald et al., 2006). The CAST captures behaviors associated with autism (e.g., differences in communication, social interaction, and repetitive behaviors and restricted interests) and has been used in large‐scale studies with teachers. Supporting Information S1: Appendix 2 contains scoring information for the CBCL TRF (Table S1–S2) and CAST (Table S3–S4).

#### Mentalizing

Children completed the Silent Film Task (Devine & Hughes, 2013), Strange Stories Task (Happé, 1994), and Triangles Task (Castelli et al., 2000) to measure mentalizing. These tasks are sensitive to individual differences in mentalizing in 8‐ to 14‐year‐old children and show excellent test–retest reliability in whole‐class testing (Devine & Hughes, 2016). The validity of these tasks is supported by longitudinal data showing that performance on standard false belief tasks at the start of primary school predicts later performance on each task at the end of primary school (Devine et al., 2016).

In the *Silent Film Task* (Devine & Hughes, 2013) children watched short film clips depicting instances of deception, misunderstanding, and false belief. Children responded to a single question about each clip, which required an explanation of a character's behavior. Children received 2 points for accurate mentalizing, 1 point for partially correct responses, and 0 points for inaccurate or irrelevant responses (Devine et al., 2023). In the *Strange Stories Task* (Happé, 1994), the researcher read aloud five short vignettes, involving deception, misunderstanding and double bluff. Children answered an open‐ended question about the characters' behavior. Accurate mentalizing received 2 points, partially correct responses received 1 point, and inaccurate responses received 0 points. In the *Frith‐Happé Triangles Animation Task* (Castelli et al., 2000), children watched three short animations, each featuring interactions between two triangles involving instances of ‘sneaking’, ‘pretending’ and ‘tricking’. Children's descriptions of the clips were rated for intentionality attribution (a score from 0 to 5) and appropriateness (a score from 0 to 2). Scores were summed for each response giving a score of 0–7 points for each clip. Inter‐rater reliability (Krippendorf's α) for coding each item ranged from 0.85 to 0.1.00 for the Strange Stories items and 0.87 to 1.00 for the SFT items. Intra‐class correlations for Triangles task Intentionality ratings ranged from 0.82 to 0.99 and from 0.74 to 0.76 for Appropriateness ratings. Supporting Information S1: Appendix 3 contains scoring and factor analysis information.

#### Social adjustment

Children rated each other using the Sociability and Leadership Scale of the *Revised Class Play* (Masten et al., 1985). Children chose one person in their class who they thought would be best at playing each of 15 ‘roles’ in a play (e.g., ‘someone who plays fair’). The number of nominations received was summed and standardized within classroom (α = 0.79). To measure *Peer Social Preference*, children nominated up to three children who they ‘most like’ and ‘least like’ to spend time with (Coie et al., 1982). The total number of nominations received by each child was standardized within classroom. Social preference, the degree to which a child is liked by their peers, was calculated by subtracting classroom standardized ‘least like’ nominations from standardized ‘most like’ nominations. Teachers completed the *Peer Social Maturity Scale* (Peterson et al., 2007) by rating children's social skills (e.g., assertion, leadership, coping with peers, understanding others' needs). High scores indicated better peer social interaction skills. Item scores were averaged (α = 0.92).

#### Covariates

Children completed group‐administered direct assessments of EF (Obradović et al., 2018). In the *Digit Span Backwards Task* (Obradović et al., 2018), children viewed a sequence of numbers displayed one at a time and then had to type the sequence in reverse order. Accuracy across 8 trials was measured by summing the correctly recalled sequences. The *Hearts and Flowers Task* (Davidson et al., 2006) consisted of two blocks. In the Hearts (control) condition, children pressed a key on the same side as a heart appearing on their screen. In the Flowers (inhibition) condition, children pressed the key on the opposite side to a flower appearing on their screen. In the *Fish Flanker Task* (Rueda et al., 2004) children were required to ‘feed the fish’ in the middle while ignoring fish on either side. In congruent trials all fish faced in the same direction. In incongruent trials children had to ignore the surrounding fish because the fish in the middle faced in the opposite direction to the other fish. In both tasks, we calculated the rate correct score (i.e., total correct trials/second) for each condition by summing the number of correct trials in each condition and dividing this by the total time (i.e., the sum of all reaction times). The EF factor is described in Supporting Information S1: Appendix 4.

Children completed the multiple‐choice section of the *Mill Hill Vocabulary Scale* (Rust, 2008) to measure verbal ability. Children selected a synonym for 20 target words and received 1 point for each correctly identified word. Total scores were age‐standardized.

Children completed an *Emotion Recognition* task (Dadds et al., 2018) using 30 images from the *Developmental Emotional Faces Stimuli Set* (Meuwissen et al., 2017). Children viewed images of faces and indicated whether the face was happy, sad, angry, fearful, or neutral. The number of correct responses for each emotion was calculated with scores ranging from 0 to 6. See Supporting Information S1: Appendix 5 for factor analysis results.

### Analysis strategy

We conducted latent variable modeling in *Mplus* Version 8 (Muthèn & Muthèn, 2017). We estimated models using a mean‐ and variance‐adjusted weighted least squares estimator (WLSMV) (Roos & Bauldry, 2022). We specified single‐level models using the ‘Type = Complex’ option in *Mplus* to adjust the standard errors and chi square statistic to account for the nesting of children within classrooms. Information about missing data is included in Supporting Information S1: Appendix 6 (Table S8). We evaluated model fit using three criteria: a root mean square error of approximation (RMSEA) of < 0.08, a comparative fit index (CFI) of > 0.90, and a Tucker Lewis Index (TLI) of > 0.90 (Roos & Bauldry, 2022). We reported the Benjamini‐Hochberg adjusted *p*‐values (*q* values) to control for the false discovery rate at 5% (Benjamini & Hochberg, 1995). When *q* values were <0.05, the test was statistically significant.

#### Preliminary analyses

**FIGURE 1:**
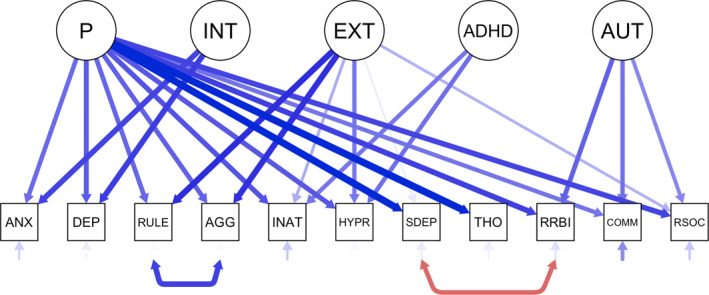
Simplified path diagram showing best‐fitting model of mental health and neurodevelopmental condition symptoms. AUT = Autism‐Specific Factor; ADHD = Attention Deficit/Hyperactivity‐Specific Factor; AGG = Aggression; ANX = Anxious/Depressed; COMM = Communication Skills; DEP = Withdrawn/Depressed; EXT = Externalizing‐specific Factor; HYPR = Impulsivity/Hyperactivity; INAT = Inattention; INT = Internalizing‐Specific Factor; P = P Factor; RULE = Rule Breaking; RRBI = Restricted Repetitive Behaviors and Interests; RSOC = Reciprocal Social Interactions; SDEP = Social Dependence; THO = Thought Problems. Blue lines indicate positive values and red lines indicate negative values. Line thickness corresponds to the strength of effect.

An orthogonal bifactor model with one general ‘P‐factor’ and four P‐free symptom factors (i.e., internalizing, externalizing, attention deficit/hyperactivity traits, and autism traits) (Figure 1) provided the best‐fitting solution (Supporting Information S1: Appendix 2, Table S5). The P‐factor captured covariance in symptoms that cut across condition‐specific subscales. The P‐free symptom factors captured variance that was not shared by the P‐factor and was unique to internalizing, externalizing, attention deficit/hyperactivity traits, and autism traits. Further preliminary analysis focused on data reduction using confirmatory factor analysis (CFA) to estimate latent factor scores for mentalizing (Supporting Information S1: Table S6, S7, Figure S1), EF, emotion recognition and social adjustment (Supporting Information S1: Appendix 3, 4, 5 and 7).

#### Main analyses

Following the pre‐registered plan, to establish the specificity of the relations between mentalizing, mental health and neurodevelopmental symptoms, we tested a model in which mentalizing was regressed onto the P‐factor and four P‐free symptom factors (Supporting Information S1: Appendix 8 for analysis code). We adjusted the estimates by regressing mentalizing onto EF, verbal ability and emotion recognition as well as age, gender, free school meals, and ethnic minority status. The independent variables were free to covary.

To examine the extent to which mentalizing accounted for links between mental health and neurodevelopmental symptoms and social adjustment, we regressed a multi‐informant social adjustment latent factor onto mentalizing and each of the mental health and neurodevelopmental latent factors (Supporting Information S1: Appendix 8 for analysis code). To isolate unique associations with social adjustment, we regressed social adjustment onto EF, verbal ability, emotion recognition, age, gender, free school meals, and ethnic minority status. The independent variables were free to covary.

## RESULTS

### Descriptive statistics

Table 1 shows the participant characteristics and descriptive statistics for all study variables. Correlations between study variables are reported in Table 2. Mentalizing was correlated with all four first‐order symptom factors (i.e., internalizing, externalizing, attention deficit/hyperactivity traits, and autism traits). In contrast, of the four P‐free symptom factors, mentalizing was only correlated with P‐free attention deficit/hyperactivity factor and P‐free autism factors. Likewise, social adjustment was correlated with each of the first‐order symptom factors. Only one of the P‐free symptom factors (i.e., P‐free attention deficit/hyperactivity) was correlated with social adjustment. The P‐factor was moderately correlated with mentalizing and strongly correlated with social adjustment.

**TABLE 2:** WLSMV estimated correlations between latent variables and covariates.

		1	2	3	4	5	6	7	8	9	10	11	12	13
1	P factor	‐												
2	Internalizing	0	‐	0.35***	0.39***	0.50***	−0.19**	−0.36***	−0.06	−0.04	−0.11*	0.02	0.02	0.11*
3	Externalizing	0	0	‐	0.74***	0.46***	−0.11*	−0.48***	−0.09*	−0.11*	−0.13**	0.05	−0.03	0.15***
4	ADHD	0	0	0	‐	0.54***	−0.27***	−0.60***	−0.17***	−0.22***	−0.26***	0.08	−0.07	0.15***
5	Autism	0	0	0	0	‐	−0.26***	−0.51***	−0.06	−0.10	−0.20***	0.02	0.01	0.15**
6	Mentalizing	−0.22***	−0.07	0.08	−0.17*	−0.15*	‐							
7	Social adjustment	−0.68***	0.06	0.02	−0.12*	−0.05	0.57***	‐						
8	Emotion recognition	−0.10*	−0.002	−0.03	−0.16**	0.01	0.41***	0.30***	‐					
9	Executive function	−0.13*	0.04	−0.03	−0.22***	−0.01	0.49***	0.37***	0.46***	‐				
10	Verbal ability	−0.18***	−0.005	0.003	−0.22***	−0.11*	0.38***	0.48***	0.30***	0.38***	‐			
11	Age	0.05	−0.01	0.02	0.06	−0.03	0.33***	0.03	0.20***	0.33***	0.01	‐		
12	Gender	0.01	0.02	−0.05	−0.13*	0.01	−0.30***	−0.20***	−0.19***	−0.07	0.01	−0.22**	‐	
13	Free school meals	0.14**	0.03	0.06*	0.07	0.08	−0.04	−0.16***	−0.02	−0.04	−0.11**	0.04	−0.07	‐
14	Ethnic minority	0.08	−0.007	0.02	0.04	0.12*	0.05	−0.08*	0.02	−0.05	−0.11**	0.08	−0.09	0.23***

*Note*: The P‐free mental health and neurodevelopmental condition factors were specified as orthogonal (i.e., the correlation between the general and specific factors was set to 0). Correlations involving P‐free condition‐specific factors are shown below the diagonal. Correlations for first‐order symptom factor scores for each condition are shown above the diagonal.

***p < 0.001, **p < 0.01, *p < 0.05.

### Relations between mentalizing and mental health and neurodevelopmental symptoms

**FIGURE 2:**
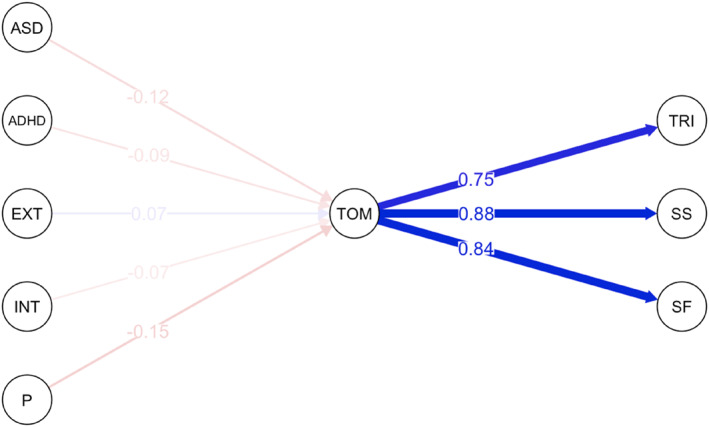
Simplified path diagram showing completely standardized estimates for the relations between mental health and neurodevelopmental symptom factors and mentalizing controlling for executive function, verbal ability, emotion recognition, age, gender, free school meal status, and ethnic minority status. ADHD = attention deficit/hyperactivity‐specific factor; AUT = autism‐specific factor; EXT = externalizing‐specific factor; INT = internalizing‐specific factor; P = P factor; SFT = silent film task; SST = strange stories task; TOM = theory of mind (mentalizing); TRIT = triangles task. Blue lines indicate positive values and red lines indicate negative values. Model Fit: *χ* ^2^ (390) = 486.425, comparative fit index = 0.929, Tucker Lewis Index = 0.910, root mean square error of approximation = 0.016, 90% CI [0.011, 0.020].

The model where mentalizing was regressed onto the P‐factor and P‐free factors provided an acceptable fit to the data (Figure 2). The P‐factor was negatively associated with mentalizing, β = −0.154, 95% CI [−0.219, −0.089], *p* = 0.0001, *q* = 0.0004, even when covariates were considered. Once the P‐factor was considered, there were no unique effects of the P‐free Internalizing factor, β = −0.072, 95% CI [−0.147, 0.002], *p* = 0.056, *q* = 0.096, or the P‐free Externalizing factor, β = 0.073, 95% CI [−0.019, 0.165], *p* = 0.120, *q* = 0.160. There were associations between the P‐free attention deficit/hyperactivity factor and mentalizing, β = −0.092, 95% CI [−0.175, −0.009], *p* = 0.030, *q* = 0.060, and between the P‐free autism factor and mentalizing, β = −0.122, 95% CI [−0.229, −0.015], *p* = 0.025, *q* = 0.060. However, these associations did not survive correction for the false discovery rate as indicated by *q* values.

### Mentalizing, mental health and neurodevelopmental symptoms, and children's social adjustment

**FIGURE 3:**
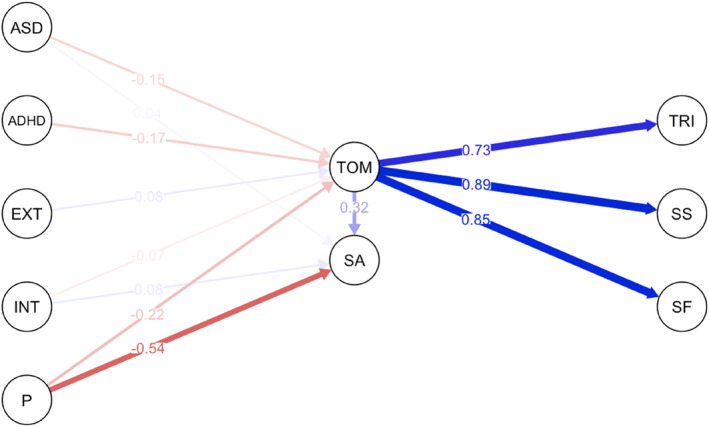
Simplified path diagram showing completely standardized estimates for the relations between mental health and neurodevelopmental symptom factors, mentalizing and social adjustment controlling for executive function, verbal ability, emotion recognition, age, gender, free school meal status, and ethnic minority status. ADHD = attention deficit/hyperactivity‐specific factor; AUT = autism‐specific factor; EXT = externalizing‐specific factor; INT = internalizing‐specific factor; P = P factor; SA = social adjustment latent factor; SFT = silent film task; SST, strange stories task; TOM = theory of mind (mentalizing); TRIT = triangles task. Blue lines indicate positive values and red lines indicate negative values. Model Fit: *χ* ^2^ (472) = 586.890, comparative fit index = 0.937, Tucker Lewis Index = 0.920, root mean square error of approximation = 0.015, 90% CI [0.011, 0.019].

The model where social adjustment was regressed onto mentalizing, the P‐factor, P‐free factors and covariates provided an acceptable fit to the data (Figure 3). Social adjustment was positively associated with children's mentalizing, β = 0.324, 95% CI [0.149, 0.500], *p* = 0.0001, *q* = 0.0004, and negatively associated with the P‐factor, β = −0.539, 95% CI [−0.623, −0.456], *p* = 0.0001, *q* = 0.0004. In contrast, the P‐free Internalizing factor, β = 0.077, 95% CI [−0.015, 0.170], *p* = 0.102, *q* = 0.1530, and P‐free Externalizing factor, β = −0.008, 95% CI [−0.120, 0.104], *p* = 0.885, *q* = 0.9240, were not associated with social adjustment. There were no unique associations between social adjustment and the P‐free attention deficit/hyperactivity factor, β = −0.005, 95% CI [−0.116, 0.105], *p* = 0.924, *q* = 0.9240, or P‐free autism factor, β = 0.036, 95% CI [−0.071, 0.143], *p* = 0.510, *q* = 0.6120. There was a significant indirect association between the P‐factor and social adjustment via mentalizing, β = −0.072, 95% CI [−0.116, −0.028], *p* = 0.001, *q* = 0.003. Mentalizing uniquely accounted for 11.76% of the association between the P‐factor and children's social adjustment.

### Sensitivity analyses

We carried out three unplanned sensitivity analyses to test the robustness of our results. First, to rule out informant effects (i.e., teachers reported on children's mental health and social maturity), we re‐ran the final model omitting the teacher‐rated social maturity indicator from the social adjustment latent factor. The model provided a good fit to the data and the pattern of results were similar (Supporting Information S1: Appendix 9.1). Second, to examine whether the effects of the P‐factor on mentalizing and social adjustment were driven by specific sets of symptoms (e.g. autism symptoms), we estimated the final model omitting each specific set of symptoms from the P‐factor model one set at a time. The pattern of unique associations between the P‐factor, mentalizing and social adjustment remained the same indicating that effects were not driven by any one set of symptoms (Supporting Information S1: Appendix 9.2, Table S9).

Third, in line with recent recommendations by Caspi et al. (2024) to aid interpretation of the P‐free factors, we specified an alternative set of models using the first‐order symptom factors. When modeled separately, each first‐order factor was negatively and significantly associated with both mentalizing and social adjustment (Supporting Information S1: Appendix 9.3, Table S10). However, when all first‐order symptom factors were entered into the model simultaneously, the pattern of associations with mentalizing and social adjustment was similar to that observed in the P‐factor model (Supporting Information S1: Appendix 9.3, Table S11–S12).

## DISCUSSION

The aim of this study was to investigate the degree to which mentalizing (or ‘theory of mind’) was associated with specific symptoms or a crosscutting dimension of mental health and neurodevelopmental symptoms in school‐aged children and the extent to which mentalizing accounted for relations between mental health and neurodevelopmental symptoms and social adjustment. We carried out a pre‐registered multi‐informant cross‐sectional study using a dimensional approach to investigate the links between mentalizing, mental health and neurodevelopmental symptoms, and social adjustment in a community sample of 1020 8‐ to 13‐year‐old children. Mentalizing was associated with a general vulnerability for mental health and neurodevelopmental conditions (i.e., P‐factor) rather than any specific set of symptoms. Difficulties with peer relationships and social interactions were transdiagnostic and the link between mental health and neurodevelopmental symptoms and social adjustment was partly accounted for by mentalizing.

### Are mentalizing difficulties in school‐aged children transdiagnostic?

Mentalizing difficulties transcended symptoms of traditional mental health and neurodevelopmental conditions. Once the general tendency for mental distress and neurodevelopmental traits (i.e., the P‐factor) was considered, P‐free symptom factors (i.e., internalizing, externalizing, attention deficit/hyperactivity, autism) did not explain any variance in mentalizing. In contrast, the sensitivity analysis showed that each of the four first‐order symptom factors was associated with mentalizing. Together these results support claims that mentalizing difficulties are transdiagnostic (Cotter et al., 2018) and break new ground by disentangling first‐order symptom factors from general psychopathology. The results also suggest that reliance on first‐order symptom factors (e.g., internalizing, externalizing) without considering other co‐occurring symptoms may generate misleading results about the specificity of difficulties with mentalizing.

Rather than representing a distinguishing feature of any specific family of symptoms, mentalizing difficulties may arise from a general vulnerability to mental health and neurodevelopmental conditions. Since case‐control studies have not previously accounted for variation in the P‐factor, studies showing differences between cases and controls on mentalizing tasks may have been driven entirely or in part by differences on this unmeasured dimension (Brislin et al., 2022). The orthogonality of the P‐factor and the P‐free symptom factors (i.e., internalizing, externalizing, attention deficit/hyperactivity, autism traits) gives rise to testable hypotheses for future research involving participants who experience clinical levels of symptoms. Our models indicate that children with high levels of autism traits and low P‐factor scores should exhibit equivalent levels of mentalizing as other children with similar levels of P but lower levels of autism traits. If replicated in samples with children and young people with autism diagnoses, this could provide new insight about ‘intact’ mentalizing among autistic people (Scheeren et al., 2013).

The current study contributes to understanding the P‐factor. Caspi and Moffitt (2018) have speculated that the P‐factor captures a tendency to experience unpleasant affect, poor impulse control, and disordered thinking. Our findings suggest the P‐factor may also capture difficulties with social cognition. P‐factor scores were uniquely associated with mentalizing even when children's EF, verbal ability, and emotion recognition were considered. While not synonymous with mentalizing, the P‐factor might also be characterized by a tendency to misunderstand others' minds. Alternatively, the P‐factor might act as a distinct risk factor because poor cognitive control and emotionality might disrupt children's developing ability to reason about others' minds (Devine et al., 2016). Longitudinal studies are needed to establish whether the P‐factor is a cause or consequence of mentalizing difficulties.

### Can mentalizing explain links between mental health and neurodevelopmental symptoms and social adjustment difficulties?

It is somewhat unsurprising that P‐factor scores were linked with poor social adjustment at school because difficulties with social participation and interaction are key criteria in a wide range of conditions (Happé & Conway, 2016). That said, our study extended existing work in two ways. First, analyses using the first‐order symptom factors suggested that social adjustment was related to each domain of mental health and neurodevelopmental symptoms. In contrast P‐free symptom factors made no unique contribution to children's social adjustment once P‐factor scores were considered. Second, consistent with the social account, mentalizing partly accounted for the relations between the P‐factor and social adjustment and relations between mentalizing and social adjustment were not explained by wider neurocognitive skills (e.g., EF or verbal ability), by other socio‐cognitive skills (e.g., emotion recognition), or by shared informant effects. If replicated among clinical samples, the results suggest that specific mental health and neurodevelopmental conditions do not automatically signal that children will experience difficulties with social adjustment. For example, our models suggest that children who score highly on autism traits, but low on the P‐factor, should exhibit similar levels of social adjustment as those who score lower on autism traits but have equivalent P‐factor scores.

Given that mentalizing was uniquely linked with social adjustment and partly accounted for the association between mental health and neurodevelopmental symptoms and social adjustment, mentalizing could provide a target for future transdiagnostic interventions to enhance children's social adjustment at school. Variation in mentalizing in school‐aged children shows unidirectional longitudinal associations with later social adjustment (Devine et al., 2016) and is amenable to change via brief classroom interventions (Lecce, 2021). Future studies are needed to test whether mentalizing interventions with children who score highly on the P‐factor improve children's social adjustment.

### Limitations

Three limitations deserve note. First, a central plank of dimensional transdiagnostic approaches is that large community samples can illuminate naturally occurring variation in and covariation between symptoms of mental health and neurodevelopmental conditions (Wise et al., 2023). One shortcoming is that variation at the upper end of each spectrum may be limited (e.g., those experiencing clinically significant levels of internalizing). The lack of significant associations between P‐free symptom factors in the current study could reflect limited variance at the upper end of the internalizing, externalizing, attention deficit/hyperactivity or autism spectra. Future work involving ‘at risk’ or clinically referred participants is needed to establish whether results generalize to those with clinically significant symptoms (Gillan & Seow, 2020). Second, the cross‐sectional design obscured the direction of associations between mental health and mentalizing and between mental health and social adjustment. However, previous work suggests mentalizing is likely to be a predictor (rather than a consequence) of social adjustment (Devine et al., 2016). Future research is needed to test whether mentalizing mediates the link between P‐factor scores and children's social adjustment. Third, while we adopted established measures of mental health and neurodevelopmental symptoms, we relied on a single informant (i.e., teachers). Future studies involving multiple informants (e.g., caregivers and teachers) and a wider range of symptoms and traits, will test the robustness of the results in different settings.

## CONCLUSIONS

The current study was the first to adopt a transdiagnostic dimensional approach to examine the relations between mentalizing (‘theory of mind’), mental health and neurodevelopmental symptoms, and social adjustment in school‐aged children. Key strengths included the use of a large, ethnically diverse community sample, a battery of reliable direct measures of mentalizing, EF, and emotion recognition, and a multi‐informant measure of children's social adjustment at school. Consistent with the transdiagnostic account, difficulties with mentalizing cut across traditional mental health and neurodevelopmental symptoms and the link between mental health symptoms and neurodevelopmental traits and social adjustment was partly accounted for by mentalizing. These findings provide new insights about the transdiagnostic nature of mentalizing difficulties and the role of mentalizing in explaining the social consequences of mental health and neurodevelopmental conditions in school‐aged children.

## AUTHOR CONTRIBUTIONS

**Rory T. Devine:** Conceptualization; data curation; formal analysis; funding acquisition; investigation; methodology; project administration; resources; supervision; writing—original draft; writing—review and editing. **Imogen Byrne:** Data curation; investigation; project administration; resources; software; writing—original draft. **Venelin Kovatchev:** Formal analysis; project administration; resources; software; writing—original draft.

## CONFLICT OF INTEREST STATEMENT

The authors declare no conflicts of interest.

## ETHICAL CONSIDERATIONS

Informed caregiver consent has been appropriately obtained and the University of Birmingham Science, Technology, Engineering and Mathematics Ethical Review Committee provided ethical approval (ERN 09‐048AP10) on 16th August 2019.

## Supporting information

Supporting Information S1

## Data Availability

The data that support the findings of this study are openly available from the University of Birmingham UBIRA: https://doi.org/10.25500/edata.bham.00001168.
