# Supplementary material for: Mentalizing difficulties are transdiagnostic and explain links between mental health and neurodevelopmental symptoms and social adjustment in school‐aged children
Source: JCPP Adv. 2025 Aug 1;6(2):e70034. doi: 10.1002/jcv2.70034 (PMC13260679; doi:10.1002/jcv2.70034)
Supplement: Supplementary file 1 — Supporting Information S1 [file JCV2-6-e70034-s001.docx]

**Mentalizing difficulties are transdiagnostic and explain links between mental health and neurodevelopmental symptoms and social adjustment in school-aged children.**

**Supporting Information**

**Contents**

| Appendix | Description | Page |
| --- | --- | --- |
| 1.0 | Power Analysis | 2 |
| 2.0 | Confirmatory Factor Analysis of Teacher Ratings of Children’s Mental Health and Neurodevelopmental Diversity | 3 |
| 3.0 | Confirmatory Factor Analysis of Children’s Mentalizing Test Performance | 12 |
| 4.0 | Confirmatory Factor Analysis of Executive Function Tasks | 17 |
| 5.0 | Confirmatory Factor Analysis of the Emotion Recognition Task. | 18 |
| 6.0 | Missing Data | 19 |
| 7.0 | Confirmatory Factor Analysis of Social Adjustment | 22 |
| 8.0 | Data Analysis Code | 23 |
| 9.0 | Sensitivity Analysis | 29 |
| 10.0 | References | 35 |

The pre-registration, study materials and data are available on the [Open Science Framework](https://osf.io/8x73r/?view_only=ac29c393379747b5906938fd744cc4ae).

1. **Power Analysis**

Sample size was based on a Monte Carlo simulation with 2000 replications carried out in *Mplus* Version 8 (Muthèn & Muthèn, 2017). The simulation included 5 latent factors: a bifactor model of mental health; a theory of mind factor (Devine & Hughes, 2016); and a social adjustment factor. Standardized direct effects were set to .25 (a medium effect) and indirect effects between P-Factor and social adjustment via mindreading were set to .06. A sample size of 1000 participants provided sufficient power to detect significant unique direct and indirect effects of mental health on Theory of Mind (.76 - .99) and Social Adjustment (.90 - .95).

**2.0. Confirmatory Factor Analysis of Teacher Ratings of Children’s Mental Health and Neurodevelopmental Symptoms**

Participants were a community-based sample recruited through schools. Using the standard scoring criteria (i.e., summed scores for each subscale), we compared the participants in our sample against established norms for the CBCL TRF Syndrome Scales (Achenbach & Rescorla, 2013). Table S1 shows the proportion of boys and girls falling in the ‘normal’, ‘borderline’, and ‘clinical’ ranges for each syndrome scale. There are no suggested cut-off scores for the CAST.

Following previous studies (Harden et al., 2020), we recoded the items of the CBCL Teacher Report Form and CAST into binary scores (i.e., not true vs somewhat or very true). Next, we inspected the tetrachoric correlations between items and aggregated those items that exhibited high correlations (>.75) and recoded these as binary items (Achenbach & Rescorla, 2013). We used CFA with the WLSMV estimator in *Mplus* Version 8 (Muthèn & Muthèn, 2017) to examine the latent factor structure of each scale in the CBCL Teacher Report Form. We scaled each latent factor using the lead indicator. We evaluated model fit using the same three standard criteria reported above (Brown, 2015). Table S2 shows the model fit indices and key features of each of these models and the omega reliability statistic for each latent factor. We saved the factor scores from this model and used these in subsequent analyses. Following others (Harden et al., 2020), we adjusted these scores by regressing the scores onto age and gender and used the residualized scores in our analyses.

The latent factor structure of the CAST was also examined. We compared the fit of three models based on the standard scoring guidelines (Ronald et al., 2008). Specifically, we examined the fit of a one factor model where all items loaded onto one latent factor, a two-factor model where restricted, repetitive behaviours and interests items loaded onto one factor and items about social-communicative skills loaded onto a second correlated factor, and a three-factor model where items for each trait loaded onto separate but correlated latent factors representing restricted, repetitive behaviours and interests, social relationships, and communication skills. None of these models provided a good fit to the data (Table S3).

We carried out a categorical data exploratory factor analysis (EFA) using the WLSMV estimator with oblique Geomin rotation to estimate a first-order factor solution incorporating between 1 and 4 latent factors. A four-factor solution provided the best fit to the data, $\chi^{2}$ (87) = 131.48, CFI = 0.982, TLI = 0.968, RMSEA = 0.026. We used the parameter estimates from this solution to specify and estimate a CFA. The model provided an adequate fit to the data (Table S3) but one latent factor was not correlated with the others. This latent factor was comprised of items about the importance of others (e.g., ‘cares how s/he is perceived by group’, ‘important to this pupil to fit in with peer group’, ‘people are important to him/her’). We therefore removed this latent factor from the model. The remaining latent factors captured individual differences in ‘restricted, repetitive behaviors’ ($\omega= .75)$, ‘reciprocated social behavior’ ($\omega= .60)$and ‘communication skills’ ($\omega= .71)$ (Table S4). We saved the factor scores from this model and used these in subsequent analyses. We used the age and gender residualized scores in our analyses.

**Latent Factor Structure of Mental Health and Neurodevelopmental Symptoms**

We compared a set of competing models based on previous research using the CBCL and approaches to modelling the mental health (e.g., Caspi et al., 2024). We used CFA with a robust maximum likelihood estimator in *Mplus* Version 8 (Muthèn & Muthèn, 2017) to estimate latent factor scores. We compared non-nested models using standard model fit indices (Brown, 2015) alongside the AIC and sample-size adjusted BIC, selecting the model with the lowest AIC and SABIC values (Caspi et al., 2024). We inspected the modification indices and respecified models to improve fit, where justifiable. To handle Heywood cases, we set the variance of items with negative residuals to close to 0 (Chen et al., 2001). To aid estimation of the higher-order and bifactor models, we set the scale of the latent factors by freely estimating the first factor loading and setting the latent factor variances to 1 (Geiser, 2013). Model fit statistics are shown in Table S5.

In the one factor model, all indicators loaded onto a single latent factor. In the three-factor model, mental health symptoms loaded onto first-order internalizing (i.e., anxious, withdrawn) and externalizing latent factors (i.e., rule breaking, aggression) and indicators of neurodevelopmental symptoms (e.g., thought problems, social dependence, inattention, hyperactivity, restricted behaviours and interests, communication, reciprocal social behaviour) loaded onto a separate first-order factor. In the five-factor model, all indicators loaded onto separate correlated first-order factors representing internalizing (i.e., anxious, withdrawn), externalizing (i.e., rule breaking, aggression), thought problems (i.e., thought problems, social dependence), attention deficits/hyperactivity (i.e., inattention, hyperactivity), and autism traits (i.e., restricted behaviours and interests, communication, reciprocal social behaviour). In the higher-order model, each of the five first-order factors loaded onto a higher-order P-Factor. In the bifactor model, all indicators loaded onto a general P-Factor and onto five P-Free symptom factors. Each factor was specified as orthogonal (i.e., the correlations between all factors were set to 0). Finally, we estimated an orthogonal S-1 bifactor model (Heinrich et al., 2023), where all indicators (except thought problems and social dependence) loaded onto both the P-Factor and four P-free latent factors (i.e., internalizing, externalizing, attention deficit/hyperactivity, autism traits). The orthogonal S-1 bifactor model with 4 P-free factors was selected as the best fitting model. The model-based estimate of reliability, $\omega_{H}$= 0.79, indicated that 79% of the variance in total scores across the original scales was accounted for by the P-Factor. Model-based estimates of reliability indicated that once variance in P was taken into account, the percentage variance in subscale scores accounted for by P-free symptom factors was 56% for internalizing, $\omega_{HS}$ = 0.56, 29% for externalizing, $\omega_{HS}$ = 0.29, 30% for attention deficit/hyperactivity, $\omega_{HS}$ = 0.30, and 35% for autism, $\omega_{HS}$ = 0.35 (Rodriguez et al., 2016).

Table S1. *Percentage of Children in ‘Borderline’ and ‘Clinical’ range based on* *CBCL TRF Syndrome Cut-off Ratings*

|  | Whole Sample (*N* = 782) | | |  | Girls (*N* = 428) | | |  | Boys (*N* = 354) | | |
| --- | --- | --- | --- | --- | --- | --- | --- | --- | --- | --- | --- |
|  | Normal | Borderline | Clinical |  | Normal | Borderline | Clinical |  | Normal | Borderline | Clinical |
| Anxious/Depressed | 87.7 | 6.9 | 5.4 |  | 86.7 | 8.6 | 4.7 |  | 89.0 | 4.8 | 6.2 |
| Withdrawn | 94.8 | 4.0 | 1.2 |  | 96.0 | 3.3 | 0.7 |  | 93.2 | 5.1 | 1.7 |
| Social Dependence | 92.4 | 3.5 | 4.1 |  | 93.0 | 3.5 | 3.5 |  | 91.8 | 3.4 | 4.8 |
| Thought Problems | 92.4 | 2.6 | 5.0 |  | 93.9 | 2.1 | 4.0 |  | 90.7 | 3.1 | 6.2 |
| Inattention | 93.6 | 3.5 | 2.9 |  | 95.8 | 2.8 | 1.4 |  | 91.0 | 4.2 | 4.8 |
| Impulsivity | 93.2 | 3.3 | 3.5 |  | 95.1 | 2.1 | 2.8 |  | 91.0 | 4.8 | 4.2 |
| Rule Breaking | 92.2 | 4.6 | 3.2 |  | 93.7 | 3.3 | 3.0 |  | 90.4 | 6.2 | 3.4 |
| Aggression | 91.9 | 4.0 | 4.1 |  | 93.7 | 3.5 | 2.8 |  | 89.8 | 4.5 | 5.6 |

Table S2. Model Fit Statistics and Summary of Parameter Estimates for CBCL Teacher Report Form Scales.

| Model | $\boldsymbol{\chi}^{\boldsymbol{2}}$ | **df** | **RMSEA** | **CFI** | **TLI** | Min. Loading | Max. Loading | $\omega$ |
| --- | --- | --- | --- | --- | --- | --- | --- | --- |
| Internalizing Latent Factors  Anxious/Depressed  Withdrawn/Depressed | 583.943 | 169 | 0.056 | 0.945 | 0.938 | .54  .49 | .89  .91 | .86  .73 |
| Externalizing Latent Factors  Rule Breaking  Aggressive Behavior | 606.894 | 298 | 0.036 | 0.990 | 0.989 | .40  .65 | .97  .98 | .80  .94 |
| Other Problems  Thought Problems  Social Dependence | 380.130 | 151 | 0.044 | 0.956 | 0.950 | .72  .48 | .92  .92 | .80  .79 |
| Attention Problems  Inattentive  Impulsive/Hyperactive | 1271.556 | 298 | 0.065 | 0.972 | 0.969 | .73  .72 | .97  .97 | .94  .93 |

Table S3. Model Fit Statistics and Summary of Parameter Estimates for CAST.

| Model | $\boldsymbol{\chi}^{\boldsymbol{2}}$ | **df** | **RMSEA** | **CFI** | **TLI** |
| --- | --- | --- | --- | --- | --- |
| One Factor Model | 1186.587 | 135 | 0.100 | 0.572 | 0.515 |
| Two Factor Model | 1162.064 | 134 | 0.099 | 0.581 | 0.522 |
| Three Factor Model* | 1140.950 | 132 | 0.099 | 0.589 | 0.524 |
| CFA based on EFA | 359.010 | 129 | 0.048 | 0.906 | 0.889 |
| Three Factor Model | 198.396 | 75 | 0.046 | 0.946 | 0.935 |

Note. *This model included a negative residual variance

Table S4. *Standardized* *WLSMV Parameter Estimates for Measurement Model of CAST Questionnaire.*

|  | **RRBI** | |  | **Reciprocal Social Behavior** | |  | **Communication** | |
| --- | --- | --- | --- | --- | --- | --- | --- | --- |
|  | **Est.** | **S.E.** |  | **Est.** | **S.E.** |  | **Est.** | **S.E.** |
| Memory for details | .68 | .06 |  |  |  |  |  |  |
| Interest in topics | .81 | .05 |  |  |  |  |  |  |
| Insistence on sameness | .77 | .05 |  |  |  |  |  |  |
| Repetitive movements | .89 | .06 |  |  |  |  |  |  |
| Takes things literally | .70 | .04 |  |  |  |  |  |  |
| Style of communication | .74 | .04 |  |  |  |  |  |  |
| Loses the listener | .81 | .04 |  |  |  |  |  |  |
| Eye gaze, facial expression, voice | .86 | .03 |  |  |  |  |  |  |
| Same interests as peers |  |  |  | .32 | .08 |  |  |  |
| Easy to interact with others |  |  |  | .94 | .02 |  |  |  |
| At least one good friend |  |  |  | .94 | .02 |  |  |  |
| Social appropriateness |  |  |  |  |  |  | .85 | .04 |
| Social behavior on own terms |  |  |  |  |  |  | .90 | .03 |
| Turns conversation to favorite subject |  |  |  |  |  |  | .81 | .04 |

**Note.** All loadings were statistically significant (*p* < .01).

Table S5. Fit Indices for Mental Health and Neurodevelopmental Models

|  | Model | χ^2^ | df | CFI | TLI | RMSEA | AIC | SABIC |
| --- | --- | --- | --- | --- | --- | --- | --- | --- |
| 1 | One Factor Model | 4524.081 | 44 | 0.425 | 0.281 | 0.361 | 8583.324 | 8632.374 |
| 2 | Three First-Order Factors | 1237.743 | 39 | 0.846 | 0.783 | 0.198 | 4531.827 | 4588.309 |
| 3 | Five First-Order Factors * | 475.921 | 35 | 0.943 | 0.911 | 0.127 | 3462.147 | 3524.573 |
| 4 | Higher-Order Model* | 1003.080 | 43 | 0.877 | 0.842 | 0.169 | 4169.895 | 4220.431 |
| 5 | Bifactor Model (Orthogonal)* | 917.333 | 38 | 0.887 | 0.837 | 0.172 | 4059.805 | 4117.773 |
| 6 | Bifactor Model (Orthogonal, Five Specific Factors, Cross-Loadings)* | 208.868 | 33 | 0.977 | 0.962 | 0.083 | 3111.839 | 3177.239 |
| 7 | Bifactor Model (Orthogonal, Four Specific Factors, Cross-Loadings)* | 175.256 | 33 | 0.982 | 0.970 | 0.074 | 3068.884 | 3134.284 |

Note. *Set residual variance for some items close to 0 due to presence of Heywood Cases.

**3.0. Confirmatory Factor Analysis of Children’s Mentalizing Test Performance**

We used confirmatory factor analysis (CFA) with a mean- and variance-adjusted weighted least squares estimator (WLSMV) in *Mplus* Version 8 (Woods et al., 2021) to examine the latent factor structure of the three measures of children’s mentalizing: the Silent Film Task, the Strange Stories Task, and the Triangles Task. We used the WLSMV estimator because the Silent Film Task and Strange Stories Task consisted of categorical items (i.e., fail, partial, pass). We evaluated model fit using three standard criteria: a root mean square error of approximation (RMSEA) of <.08, a comparative fit index (CFI) of >.90, and a Tucker Lewis Index (TLI) of >.90. We examined modification indices to identify areas of strain in each model and revised the model if free estimation of a given parameter was theoretically justified. Given that each indicator consisted of a single item (rather than a composite score), completely standardized loadings of .30 were considered salient factor loadings.

Drawing on previous work (e.g., Devine & Hughes, 2016), we tested a series of competing measurement models. Based on prior research (Devine et al., 2023), we permitted two residual terms to correlate in each model (i.e., Items 3 and 5 of the Silent Film Task and Items 3 and 5 of the Strange Stories Task). We estimated five competing models as follows: (1) a model where items from the Silent Film Task, Strange Stories Task and Triangles Task all loaded onto one latent factor (Model 1); (2) a model where items from the Silent Film Task and Strange Stories Task loaded onto one latent factor and items from the Triangles Task loaded onto a second correlated latent factor (Model 2); (3) a model where items from each task loaded onto three task-specific correlated latent factors (Model 3); (4) a higher-order model where three task-specific latent factors loaded onto a second-order theory-of-mind latent factor (Model 4); and (5) a bifactor model where all items loaded onto one general theory-of-mind latent factor and two task-specific latent factors representing the Strange Stories Task and Triangles Task (Model 5).

Table S6 shows item-level descriptive statistics for the mentalizing task indicators and Table S7 shows the model fit statistics for each measurement model. All models provided a good fit to the data using standard criteria. The three latent factor model provided a better fit to the data than the one- and two-latent factor models. There were strong correlations between the Silent Film Task and Strange Stories Task latent factors, *Est*. = .74, 95% CI [.58, .89], the Silent Film Task and Triangles Task latent factors, *Est*. = .68, 95% CI [.53, .82], and between the Strange Stories and Triangles Task latent factors, *Est*. = .72, 95% CI [.59, .85]. Note that, factor solutions with three correlated latent factors and higher-order factor solutions with three first-order latent factors yield the same goodness of fit indices (Brown, 2015). So, instead of comparing model fit, we examined the strength of the loadings of each latent factor onto a higher-order mentalizing latent factor. The second-order factor model showed that each task-specific factor loading strongly onto the higher-order mentalizing latent factor (see Figure S1), indicating that the mentalizing latent factor accounted for 68.9% of the variance in the Silent Film Task latent factor, 78.3% of the variance in the Strange Stories Task latent factor, and 66.1% of the variance in the Triangles Task latent factor. We selected the second-order latent factor model (Model 4) over the competing models on this basis.

Table S6. *Item-level Performance on the Mentalizing Tasks.*

|  | ***N*** | **Fail**  **(%)** | **Partial (%)** | **Pass (%)** | **M** | **SD** | **Range** |
| --- | --- | --- | --- | --- | --- | --- | --- |
| SS1. Brian’s Story | 974 | 5.7 | 74.7 | 19.5 |  |  |  |
| SS2. Mrs Peabody’s Story | 970 | 4.0 | 50.1 | 45.9 |  |  |  |
| SS3. The Prisoner’s Story | 952 | 36.7 | 22.1 | 41.3 |  |  |  |
| SS4. Simon’s Story | 966 | 8.7 | 47.3 | 44.0 |  |  |  |
| SS5. The Burglar’s Story | 960 | 40.1 | 41.8 | 18.1 |  |  |  |
| SF1. Why do the men hide? | 962 | 65.9 | 21.8 | 12.3 |  |  |  |
| SF2. What is the woman thinking? | 946 | 27.8 | 17.7 | 54.5 |  |  |  |
| SF3. Why does the driver lock Harold in the van? | 955 | 39.0 | 6.5 | 54.6 |  |  |  |
| SF4. What is the delivery man feeling and why? | 962 | 8.3 | 43.7 | 48.0 |  |  |  |
| SF5. Why did Harold pick up the cat? | 967 | 49.1 | 6.8 | 44.1 |  |  |  |
| SF6. Why did Harold fan Mildred? | 952 | 41.6 | 18.0 | 40.4 |  |  |  |
| TRI1. Sneaking | 839 |  |  |  | 5.17 | 1.59 | 0 – 7 |
| TRI2. Pretending | 826 |  |  |  | 3.27 | 1.69 | 0 – 7 |
| TRI3. Tricking | 813 |  |  |  | 4.04 | 1.60 | 0 – 7 |

*Table S7. Model Fit Statistics for Confirmatory Factor Analysis of Mentalizing Data*

|  | **Model Description** | $\boldsymbol{\chi}^{\boldsymbol{2}}$ | **df** | **RMSEA** | **CFI** | **TLI** | **Notes** |
| --- | --- | --- | --- | --- | --- | --- | --- |
| 1 | One single latent factor | 136.385 | 75 | 0.029 | 0.938 | 0.924 |  |
| 2 | Two correlated latent factors | 115.468 | 74 | 0.024 | 0.958 | 0.948 | Latent factor correlation: .76 |
| 3 | Three correlated latent factors | 104.212 | 72 | 0.021 | 0.967 | 0.959 | Latent factor correlations: .68 - .74 |
| 4 | Second-Order Factor | 104.212 | 72 | 0.021 | 0.967 | 0.959 | Second-order factor explained 66%-78% of variance in first-order factors. |
| 5 | Bifactor Model (S-1) | 100.308 | 67 | 0.022 | 0.966 | 0.954 | Non-significant loadings on SS specific factor. |

*Figure S1.*

| 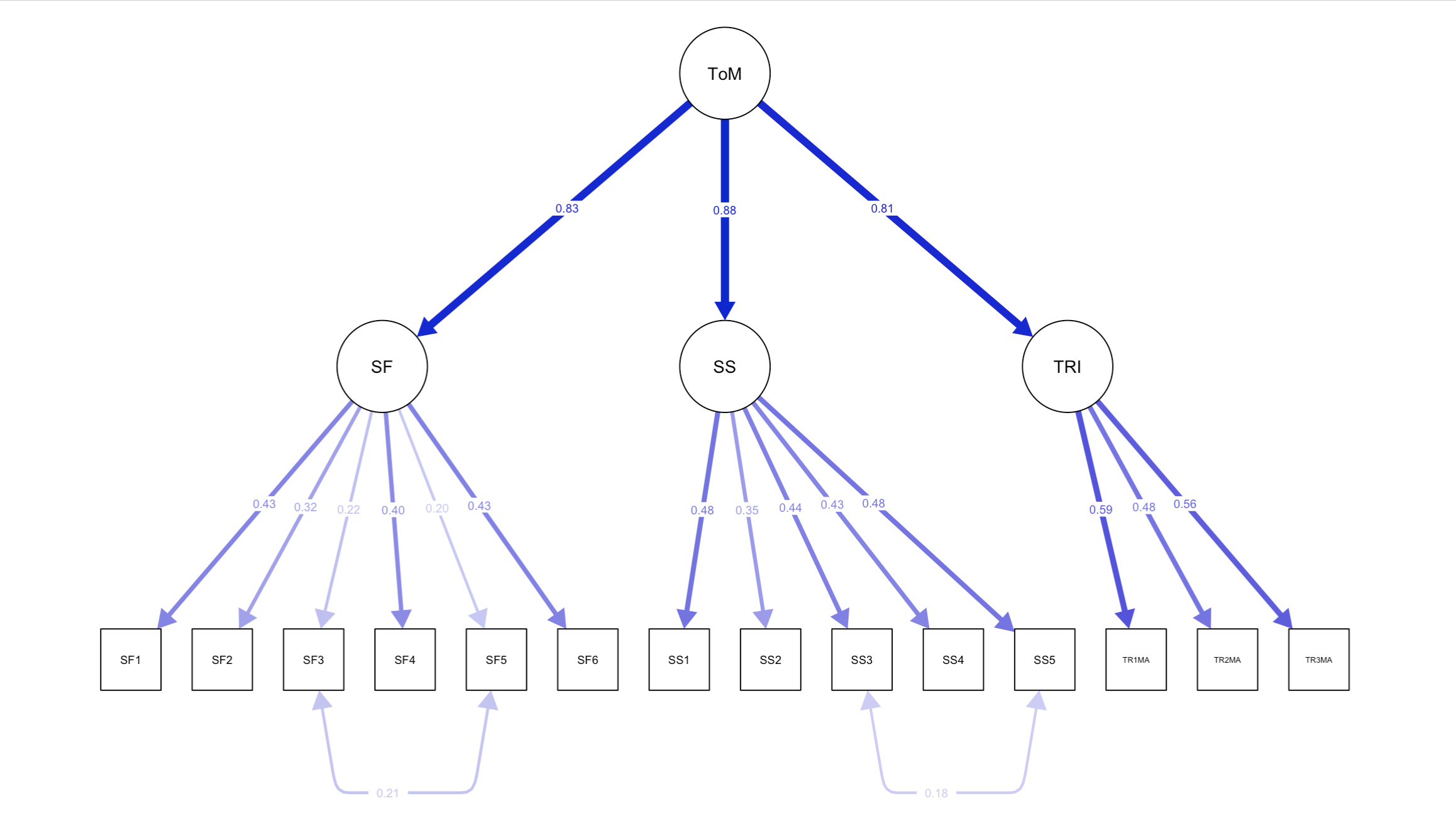 | 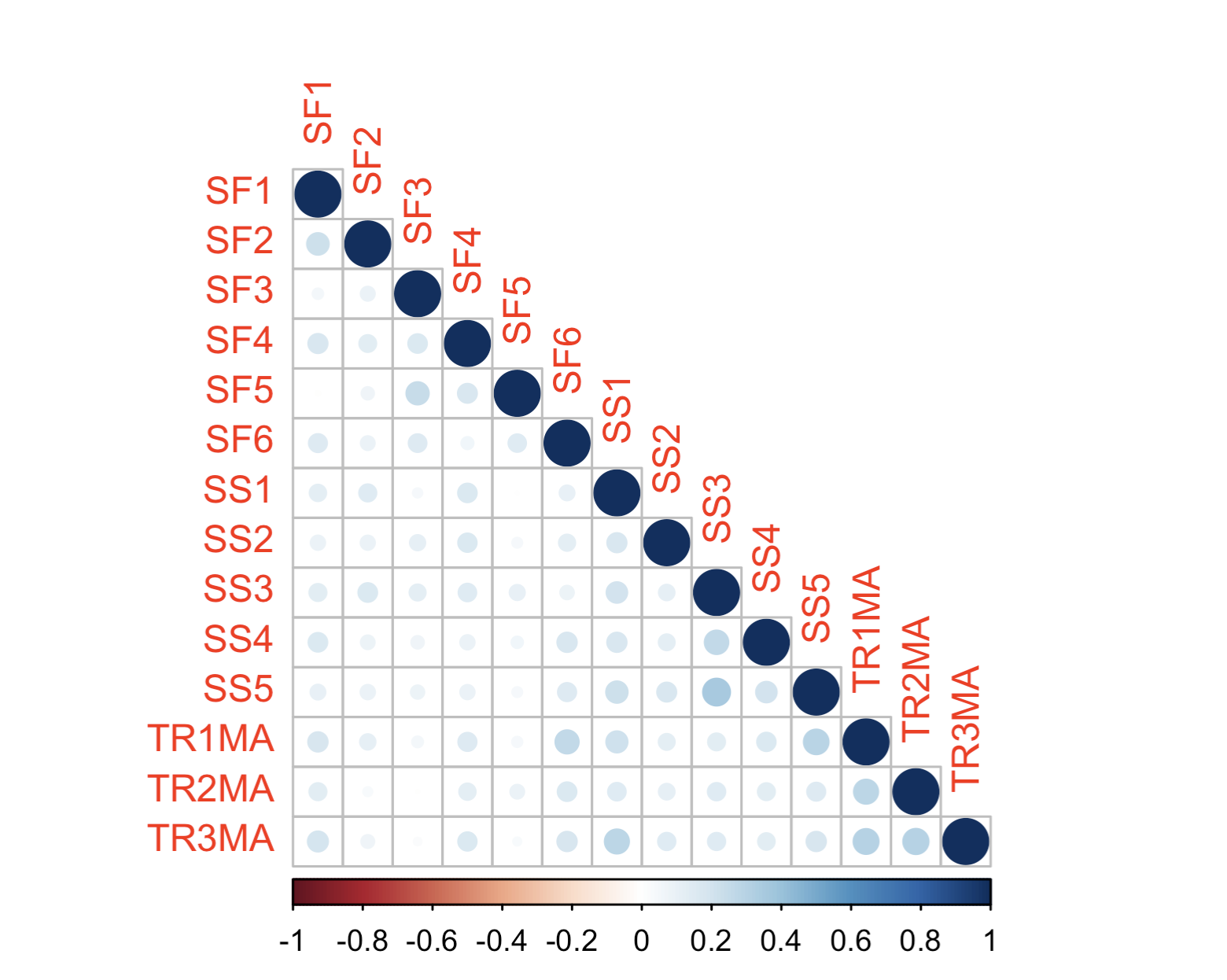 |
| --- | --- |
| Caption. Path Diagram Depicting Standardized Estimates for Second-Order Mentalizing Model and Polychoric Correlation Matrix for Mentalizing Indicators. *Note.* SF = Silent Film Task. SS = Strange Stories Task. TRI = Triangles Task. ToM = Theory of Mind/Mentalizing. | |

**4.0. Confirmatory Factor Analysis of Executive Function Tasks.**

Children performed better on congruent trials of the Fish Flanker task, *M* = 1.41, *SD* = 0.46, than on incongruent trials, *M* = 1.36, *SD* = 0.46, *t* (923) = 4.40, *p* < .001, *d* = 0.15, 95%CI [0.08, 0.21]. Likewise, performance on the no conflict trials, *M* = 2.09, *SD* = 0.68, was better than their performance on the conflict trials, *M* = 1.61, *SD* = 0.68, of the Hearts and Flowers Task, *t* (891) = 21.16, *p* < .001, *d* = 0.68, 95%CI [0.64, 0.78]. On the Backward Digit Span Task, performance declined across the task as the length of digit sequences increased, *F* (3, 2575) = 940.34, *p* < .001. There were significant differences between each trial type with 2-digit trials, *M* = 1.88, *SD* =0.39, being the easiest and 5-digit trials, *M* = 0.56, *SD* = 0.76, being the most challenging. All contrasts were significant with Cohen’s *d* ranging from 0.41, 95%CI [0.34, 0.48] to 1.63, 95%CI [1.53, 1.73].

We used CFA with a robust maximum likelihood estimator to estimate latent factor scores for executive function. Specifically, we tested a one factor model in which the incongruent trials score of the Fish Flanker Task, conflict trials score of the Hearts and Flowers Task, and total score for the Backward Digit Span Task loaded onto one latent factor. The model was just-identified as the number of freely estimated parameters equalled the number of pieces of known information in the input variance-covariance matrix (Brown, 2015). Each indicator loaded significantly on the latent factor: Hearts and Flowers Task *Std. Est.* = .65, *p* <.0001, Fish Flanker Task *Std. Est.* = .60, *p* <.0001, Backward Digit Span Task *Std. Est.* = .42, *p* <.0001. We saved the factor scores from this model and used these in subsequent analyses.

**5.0. Confirmatory Factor Analysis of the Emotion Recognition Task.**

Children’s performance on the emotion recognition task varied by the type of emotion depicted, *F* (4, 3540) = 319.584, *p* < .001. There were no significant differences in performance on sad, *M* = 4.46, *SD* = 1.35, and neutral faces, *M* = 4.49, *SD* = 1.55, *t* (885) = -0.44, *p* = .66, Cohen’s *d* = -.02, 95%CI [-0.08, 0.05]. All other contrasts were significant (*p* < .001) with children performing best on happy faces, *M* = 5.10, *SD* = 1.13, then neutral and sad faces, fearful faces, *M* = 3.82, *SD* = 1.56, and angry faces, *M* = 3.21, *SD* = 1.40, Cohen’s *d* ranged from 0.34, 95%CI [0.27, 0.41] to 1.22, 95%CI [1.13, 1.31].

We used CFA with a robust maximum likelihood estimator to generate latent factor scores for emotion recognition. Specifically, we tested a one factor model in which accuracy scores for each emotion loaded onto one latent factor. A one-factor model fit the data well, $\chi^{2}$ (4) = 14.02, CFI = 0.98, TLI = 0.94, RMSEA = 0.05. Standardized loadings ranged from .39 to .65 and all loadings were statistically significant, *p* < .0001. We saved the factor scores from this model and used these in subsequent analyses.

**6.0. Missing Data**

Of the 1020 children (93% participation rate) included in the study, 890 (87.3%) children participated in both study visits and 130 (12.7%) children participated in one study visit. Teachers completed questionnaires for 786 (77.1%) of the children. Missing teacher questionnaires were attributed to the cessation of testing due to the Covid-19 pandemic restrictions. The majority of participants (N=720, 70.6%) participated in both testing sessions and their teachers completed the questionnaire. A further 170 children (16.7%) participated in two testing sessions but their teachers did not return a questionnaire. Of the remaining children, 66 (6.5%) participated in one testing session and their teacher returned a questionnaire and 64 (6.3%) participated one testing session and their teacher did not return a questionnaire.

In total, teacher questionnaires were available for 786 children (77.1%). We examined patterns of unit missingness by testing whether the availability of a completed teacher questionnaire (0 = no questionnaire returned, 1 = questionnaire returned) was related to key study variables such as child demographic factors (i.e., age, gender) and children’s performance on tests of theory of mind and peer-rated social adjustment (e.g., Newsom, 2015). We also investigated whether missingness was related to a potential auxiliary variable (Woods et al., 2021). Specifically, we examined if availability of teacher questionnaires was related to whether children attended a school with high, average or low levels of pupils eligible for free school meals (an indicator of socio-economic status). At the time of data collection, on average 17.7% of primary school pupils and 15.9% of secondary school pupils were eligible for free school meals.

To this end, we tested a logistic regression in which the binary teacher questionnaire completion item was regressed onto age in years, gender (0 = girl, 1 = boy), free school meals, theory of mind latent factor scores and social adjustment latent factor scores. There was no link between missingness and theory of mind or social adjustment (Table S8). However, age and whether a child attended a school with high levels of deprivation both predicted teacher questionnaire completion. Specifically, teachers of older children were less likely to return a questionnaire than teachers of younger children. Teachers of children in high deprivation schools were more likely to return a completed questionnaire than those in low deprivation schools.

We concluded that the teacher questionnaires were likely to be missing at random (MAR) as the likelihood of missingness was related to age but not to our key study measures (i.e., theory of mind or social adjustment) (Woods et al., 2021). We used all available data in our models (i.e., data from all 1020 participants) (Newsom, 2024). The WLSMV estimator uses a combination of pairwise and full information maximum likelihood (FIML) to estimate parameters and is appropriate for models with categorical variables and data that are missing at random (Newsom, 2024). Given its link with teacher questionnaire missingness, we included school socio-economic status as an auxiliary variable in our analyses and age as an observed variable in our models (Newsom, 2024).

*Table S8. Logistic Regression Predicting Return of Teacher Questionnaires.*

| **Independent Variable** | **Est.** | **S.E.** | ***p*** | **OR** |
| --- | --- | --- | --- | --- |
| Age | -0.415 | 0.083 | 0.0001 | 0.661 |
| Gender (0 = Girl, 1 = Boy) | 0.260 | 0.178 | 0.144 | 1.296 |
| School SES | 2.286 | 0.276 | 0.0001 | 9.837 |
| Mentalizing | 0.023 | 0.142 | 0.872 | 1.023 |
| Social Adjustment | 0.119 | 0.108 | 0.274 | 1.126 |

Note. School SES was measured using a dummy code comparing children who attended a school with low or average levels of free school meal uptake (0) or high levels of free school meal uptake (1).

**7.0. Confirmatory Factor Analysis of Social Adjustment**

We used CFA with a robust maximum likelihood estimator to estimate social adjustment latent factor scores. Specifically, we tested a one factor model in which the scores from the Peer-assessed Revised Class Play Sociability and Leadership scale, Peer-rated Social Preference, and Teacher-rated Social Maturity loaded onto one latent factor. The model was just-identified as the number of freely estimated parameters equalled the number of pieces of known information in the input variance-covariance matrix (Brown, 2015). Each indicator loaded significantly on the latent factor: Revised Class Play *Std. Est.* = .80, *p* <.0001, Social Preference *Std. Est.* = .66, *p* <.0001, Social Maturity *Std. Est.* = .52, *p* <.0001.

**8.0. Data Analysis Code**

**8.1. Mplus Syntax for Model Examining Relations between Mentalizing and Mental Health and Neurodevelopmental Symptoms**

Title: Model 1 P-Factor and Mentalizing;

Data: File is 2024_07_WP1_Data_Share.dat;

Variable: Names are

ID !unique child ID

Part

School

CID !classroom ID

SCHFSM

SFSM !school-level free school meals

EthB !1=Majority ethnicity 2=Minoritized ethnicity

ETH

Teach

Session

Gender !0=girl 1=boy

AgeR !Age in Years

FSM !0=no free school meals 1=free school meals

EAL !0=native english speaker 1=english as additional language

SEND !0=no special educational needs 1=special educational needs

VA !Verbal Ability (age standardized)

SF1 !Silent Film Task item 1

SF2 !Silent Film Task item 2

SF3 !Silent Film Task item 3

SF4 !Silent Film Task item 4

SF5 !Silent Film Task item 5

SF6 !Silent Film Task item 6

SS1 !Strange Stories Task item 1

SS2 !Strange Stories Task item 2

SS3 !Strange Stories Task item 3

SS4 !Strange Stories Task item 4

SS5 !Strange Stories Task item 5

TR1MA !Triangles Task item 1

TR2MA !Triangles Task item 2

TR3MA !Triangles Task item 3

CBCLFSAD !CBCL TRF Anxious/Depressed Score

CBCLFSWD !CBCL TRF Withdrawn/Depressed Score

CBCLFSRB !CBCL TRF Rule Breaking

CBCLFSAB !CBCL TRF Aggressive Behaviour

CBCLFSIA !CBCL TRF Inattention

CBCLFSIH !CBCL TRF Impulsivity/Hyperactivity

CBCLFSSP !CBCL TRF Social Problems

CBCLFSTP !CBCL TRF Thought Problems

CASTFSNI !CAST Restricted Repetitive Behaviours/Narrow Interests

CASTFSCS !CAST Communication Skills

CASTFSRS !CAST Reciprocal Social Interaction

SMS_MEAN !Teacher-Rated Peer Social Maturity

SP_Z_R !Peer Nomination Social Preference Score

RCP_R !Peer-Rated Sociability-Leadership Score

ERFSCORE !Emotion Recognition Factor Score

EFFSCORE !Executive Function Factor Score

SFTOT !Silent Film Task Summed Total

SSTOT !Strange Stories Task Summed Total

TRITOT !Triangles Task Summed Total

EMRTOT !Emotion Recognition Summed Total

;

Usevar are AgeR Gender VA FSM EthB ERFSCORE EFFSCORE TR1MA TR2MA TR3MA CBCLFSAD CBCLFSWD CBCLFSRB CBCLFSAB CBCLFSIA CBCLFSIH CBCLFSSP CBCLFSTP CASTFSNI CASTFSCS CASTFSRS SF1 SF2 SF3 SF4 SF5 SF6 SS1 SS2 SS3 SS4 SS5;

Categorical are SF1 SF2 SF3 SF4 SF5 SF6 SS1 SS2 SS3 SS4 SS5;

Auxiliary = SFSM; Missing are all (-99); IDVAR = ID; Cluster = CID;

Define:

Center ager (grandmean);

Analysis:

Estimator = wlsmv;

Type = complex;

Model:

!Specify Bifactor Model

!P-Factor (General Factor)

P BY CBCLFSAD* CBCLFSWD CBCLFSRB CBCLFSAB CBCLFSIA CBCLFSIH CBCLFSSP CBCLFSTP CASTFSNI CASTFSCS CASTFSRS; P@1;

!P-Free Internalizing Factor

INT BY CBCLFSAD* CBCLFSWD; INT@1;

!P-free Externalizing Factor

EXT BY CBCLFSRB* CBCLFSAB; EXT@1;

!P-free attention deficit/hyperactivity Factor

ADHD BY CBCLFSIA* CBCLFSIH; ADHD@1;

!P-free Autism Factor

ASD BY CASTFSNI* CASTFSCS CASTFSRS; ASD@1;

!Specific Factors orthogonal to P-Factor

P with INT@0; P with EXT@0; P with ADHD@0; P with ASD@0;

!Specific Factors orthogonal with other Specific Factors

INT with EXT@0; Int with ADhd@0; Int with ASD@0; Ext with ADHD@0;

Ext with ASD@0; ADHD with ASD@0;

!set residual variance close to 0

CBCLFSIH@0.01; CBCLFSWD@0.01; CBCLFSAB@0.01; CBCLFSRB@0.01;

CBCLFSIA@0.01;

!modification to orthogonal solution

!cross loading

EXT BY CASTFSRS; EXT BY CBCLFSSP;

!correlated residual

CBCLFSAB WITH CBCLFSRB;

!Theory of Mind Latent Factor

!Silent Film Task Latent Factor

SF BY SF6* SF2 SF3 SF4 SF5 SF1; SF@1;

!Correlated Residual Term

SF3 with SF5;

!Strange Stories Task Latent Factor

SS BY SS1* SS2 SS3 SS4 SS5; SS@1;

!Triangles Task Latent Factor

TRI BY TR1MA* TR2MA TR3MA; TRI@1;

!Theory of Mind Higher Order Factor

ToM BY SF* SS TRI; ToM@1;

!Theory of Mind Regressed onto Mental Health Factors

ToM ON P Int Ext ADHD ASD;

!Adjust model for effects of covariates

!Regress Mentalizing onto covariates

ToM on ager gender va fsm EthB ERFSCORE EFFSCORE;

!Permit independent variables to correlate

p with ager gender va fsm EthB ERFSCORE EFFSCORE;

Int with ager gender va fsm EthB ERFSCORE EFFSCORE;

Ext with ager gender va fsm EthB ERFSCORE EFFSCORE;

adhd with ager gender va fsm EthB ERFSCORE EFFSCORE;

asd with ager gender va fsm EthB ERFSCORE EFFSCORE;

[ager]; [gender]; [va]; [fsm]; [ethb]; [ERFSCORE]; [EFFSCORE];

!modification to improve fit

SS3 ON VA;

SF1 ON GENDER;

Output:

sampstat; modindices (all); standardized; cinterval; tech4;

**8.2.** **Mplus Syntax for Model Examining Relations Between Mentalizing, Mental Health and Neurodevelopmental Symptoms, and Children’s Social Adjustment**

Title: Model 1 P-Factor, Mentalizing and Social Adjustment;

Data: File is 2024_07_WP1_Data_Share.dat;

Variable: Names are

ID !unique child ID

Part

School

CID !classroom ID

SCHFSM

SFSM !school-level free school meals

EthB !1=Majority ethnicity 2=Minoritized ethnicity

ETH

Teach

Session

Gender !0=girl 1=boy

AgeR !Age in Years

FSM !0=no free school meals 1=free school meals

EAL !0=native english speaker 1=english as additional language

SEND !0=no special educational needs 1=special educational needs

VA !Verbal Ability (age standardized)

SF1 !Silent Film Task item 1

SF2 !Silent Film Task item 2

SF3 !Silent Film Task item 3

SF4 !Silent Film Task item 4

SF5 !Silent Film Task item 5

SF6 !Silent Film Task item 6

SS1 !Strange Stories Task item 1

SS2 !Strange Stories Task item 2

SS3 !Strange Stories Task item 3

SS4 !Strange Stories Task item 4

SS5 !Strange Stories Task item 5

TR1MA !Triangles Task item 1

TR2MA !Triangles Task item 2

TR3MA !Triangles Task item 3

CBCLFSAD !CBCL TRF Anxious/Depressed Score

CBCLFSWD !CBCL TRF Withdrawn/Depressed Score

CBCLFSRB !CBCL TRF Rule Breaking

CBCLFSAB !CBCL TRF Aggressive Behaviour

CBCLFSIA !CBCL TRF Inattention

CBCLFSIH !CBCL TRF Impulsivity/Hyperactivity

CBCLFSSP !CBCL TRF Social Problems

CBCLFSTP !CBCL TRF Thought Problems

CASTFSNI !CAST Restricted Repetitive Behaviours/Narrow Interests

CASTFSCS !CAST Communication Skills

CASTFSRS !CAST Reciprocal Social Interaction

SMS_MEAN !Teacher-Rated Peer Social Maturity

SP_Z_R !Peer Nomination Social Preference Score

RCP_R !Peer-Rated Sociability-Leadership Score

ERFSCORE !Emotion Recognition Factor Score

EFFSCORE !Executive Function Factor Score

SFTOT !Silent Film Task Summed Total

SSTOT !Strange Stories Task Summed Total

TRITOT !Triangles Task Summed Total

EMRTOT !Emotion Recognition Summed Total

;

Usevar are AgeR Gender VA FSM EthB ERFSCORE EFFSCORE TR1MA TR2MA TR3MA CBCLFSAD CBCLFSWD CBCLFSRB CBCLFSAB CBCLFSIA CBCLFSIH CBCLFSSP CBCLFSTP CASTFSNI CASTFSCS CASTFSRS SF1 SF2 SF3 SF4 SF5 SF6 SS1 SS2 SS3 SS4 SS5 SMS_MEAN SP_Z_R RCP_R;

Categorical are SF1 SF2 SF3 SF4 SF5 SF6 SS1 SS2 SS3 SS4 SS5;

Auxiliary = SFSM; Missing are all (-99); IDVAR = ID; Cluster = CID;

Define:

Center ager (grandmean);

Analysis:

Estimator = wlsmv;

Type = complex;

Model:

!Specify Bifactor Model

!P-Factor (General Factor)

P BY CBCLFSAD* CBCLFSWD CBCLFSRB CBCLFSAB CBCLFSIA CBCLFSIH CBCLFSSP CBCLFSTP CASTFSNI CASTFSCS CASTFSRS; P@1;

!P-Free Internalizing Factor

INT BY CBCLFSAD* CBCLFSWD; INT@1;

!P-Free Externalizing Factor

EXT BY CBCLFSRB* CBCLFSAB; EXT@1;

! P-Free attention deficit/hyperactivity Factor

ADHD BY CBCLFSIA* CBCLFSIH; ADHD@1;

! P-Free Autism Factor

ASD BY CASTFSNI* CASTFSCS CASTFSRS; ASD@1;

!Specific Factors orthogonal to P-Factor

P with INT@0; P with EXT@0; P with ADHD@0; P with ASD@0;

!Specific Factors orthogonal with other Specific Factors

INT with EXT@0; Int with ADhd@0; Int with ASD@0; Ext with ADHD@0;

Ext with ASD@0; ADHD with ASD@0;

!set residual variance close to 0

CBCLFSIH@0.01; CBCLFSWD@0.01; CBCLFSAB@0.01; CBCLFSRB@0.01;

CBCLFSIA@0.01;

!modification to orthogonal solution

!cross loading

EXT BY CASTFSRS;

EXT BY CBCLFSSP;

!correlated residual

CBCLFSAB WITH CBCLFSRB;

!Theory of Mind Latent Factor

!Silent Film Task Latent Factor

SF BY SF6* SF2 SF3 SF4 SF5 SF1; SF@1;

!Correlated Residual Term

SF3 with SF5;

!Strange Stories Task Latent Factor

SS BY SS1* SS2 SS3 SS4 SS5; SS@1;

!Triangles Task Latent Factor

TRI BY TR1MA* TR2MA TR3MA; TRI@1;

!Mentalizing Higher Order Factor

ToM BY SF* SS TRI; ToM@1;

!Social Adjustment Latent Factor

SA BY SMS_MEAN* SP_Z_R RCP_R; SA@1;

!Social Adjustment Regressed onto Theory of Mind and Mental Health Factors

SA ON ToM P Int Ext ADHD ASD;

!Mentalizing Regressed onto Mental Health Factors

ToM ON P Int Ext ADHD ASD;

!Adjust model for effects of covariates

!Regress SA onto covariates

SA ON ager gender va fsm EthB ERFSCORE EFFSCORE;

!Permit independent variables to correlate

ToM with ager gender va fsm EthB ERFSCORE EFFSCORE;

p with ager gender va fsm EthB ERFSCORE EFFSCORE;

Int with ager gender va fsm EthB ERFSCORE EFFSCORE;

Ext with ager gender va fsm EthB ERFSCORE EFFSCORE;

adhd with ager gender va fsm EthB ERFSCORE EFFSCORE;

asd with ager gender va fsm EthB ERFSCORE EFFSCORE;

[ager]; [gender]; [va]; [fsm]; [ethb]; [ERFSCORE]; [EFFSCORE];

!modification to improve fit

SS3 ON VA; SF1 ON GENDER; SP_Z_R with RCP_R;

Model Indirect:

!Path from P to SA via ToM

SA IND P;

Output:

sampstat; modindices (all); standardized; cinterval; tech4;

**9.0. Sensitivity Analyses**

**9.1. Informant Effects.**

To rule out informant effects (i.e., teachers reported on children’s mental health and social maturity), we re-ran the model linking the P-Factor, mentalizing and social adjustment omitting the teacher-rated social maturity indicator from the social adjustment latent factor. The model provided an acceptable fit to the data, $\chi^{2}$ (440) = 548.835, CFI = 0.933, TLI = 0.915, RMSEA = 0.016. There were significant and unique associations between mentalizing and P-Factor scores, $\beta$ = -0.223, *p* < 0.0001, social adjustment and mentalizing, $\beta$ = 0.241, *p* = 0.005, and between social adjustment and P-Factor scores, $\beta$ = -0.343, *p* < 0.0001. There was a significant indirect association between the P-Factor and social adjustment via mentalizing, $\beta$ = -0.054, *p* = 0.008.

**9.2. P-Factor Sensitivity Analysis.**

To examine whether associations between the P-factor, mentalizing and social adjustment were driven by specific sets of symptoms (e.g. autism symptoms), we re-ran our final model omitting each set of symptoms from the P-factor model one set at a time. The model fit indices for each model are shown in Table S9. The key parameter estimates are shown in Table S10. The results show that the P-factor remained uniquely associated with mentalizing and with social adjustment even when specific sets of symptoms are removed from the model. All indirect associations were also replicated.

**9.3. First-Order Symptom Factors and P-Free Factors.**

To interpret the P-free factors we ran two sets of analyses focused on first-order symptom factors. First, we specified and tested four separate models using only one first-order symptom factor in each model (e.g., internalizing). Then, we specified and tested an adjusted model with all four first-order factors entered simultaneously. The fit of each of these models is shown in Table S11. The key parameter estimates for these models are shown in Table S12. When entered separately, first-order symptom factors showed unique associations with mentalizing and social adjustment. However, when entered simultaneously, the unique effects of first-order symptom factors on both mentalizing and social adjustment were altered. In the adjusted model, the associations between mentalizing and each first-order symptom factor were similar to the P-free factors in the P-factor model. Likewise, the pattern of associations between social adjustment and each first-order symptom factor was similar in the adjusted model and P-factor model. These results suggest that first-order symptom factors contain variance that is common across all symptom factors (i.e., the P-Factor) and that P-free factors provide estimates similar to those that control for all other symptoms (Caspi et al., 2024).

*Table S9. Model Fit Indices for P-Factor Sensitivity Analyses*

| Model | $\boldsymbol{\chi}^{\boldsymbol{2}}$ | **df** | **RMSEA** | **CFI** | **TLI** |
| --- | --- | --- | --- | --- | --- |
| Without Internalizing Items | 520.618 | 418 | 0.016 | 0.944 | 0.929 |
| Without Externalizing Items | 519.135 | 419 | 0.015 | 0.940 | 0.924 |
| Without Attention Deficit/Hyperactivity Items | 507.021 | 417 | 0.015 | 0.944 | 0.929 |
| Without Autism Items | 488.537 | 389 | 0.016 | 0.937 | 0.919 |
| Without Social Dependency | 552.166 | 441 | 0.016 | 0.934 | 0.916 |
| Without Thought Problems | 549.341 | 439 | 0.016 | 0.936 | 0.918 |

*Table S10. P-Factor Sensitivity Analysis*

|  | **P-Factor & Mentalizing** | | | **P-Factor & Social Adjustment** | | | **Indirect Effect** | | |
| --- | --- | --- | --- | --- | --- | --- | --- | --- | --- |
| **Model** | **Est.** | **95% CI** | **p** | **Est.** | **95% CI** | **p** | **Est.** | **95% CI** | **p** |
| Original Full Model | -.22 | -.33, -.11 | .0001 | -.54 | -.62, -.46 | .0001 | -.07 | -.12, -.03 | .001 |
| Without Internalizing | -.23 | -.34, -.11 | .0001 | -.54 | -.63, -.46 | .0001 | -.07 | -.12, -.03 | .002 |
| Without Externalizing | -.22 | -.33, -.11 | .0001 | -.54 | -.63, -.46 | .0001 | -.07 | -.12, -.03 | .001 |
| Without Attention Deficit/Hyperactivity | -.22 | -.33, -.12 | .0001 | -.53 | -.62, -.44 | .0001 | -.08 | -.12, -.03 | .001 |
| Without Autism | -.22 | -.33, -.11 | .0001 | -.55 | -.64, -.47 | .0001 | -.07 | -.11, -.03 | .002 |
| Without Social Dependency | -.20 | -.32, -.09 | .001 | -.50 | -.58, -.41 | .0001 | -.06 | -.11, -.02 | .003 |
| Without Thought Problems | -.21 | -.30, -.11 | .0001 | -.51 | -.59, -.44 | .0001 | -.07 | -.11, -.02 | .002 |

*Note.* The table shows the unique association between the P-Factor and mentalizing, the P-Factor and Social Adjustment, and the Indirect Effect of the P-Factor on Social Adjustment Via Mentalizing in the Original Full Model (including all symptom indicators) and the results of each model with one set of symptom indicators omitted.

*Table S11. Model Fit Indices for First-Order Factor Models*

| Model | $\boldsymbol{\chi}^{\boldsymbol{2}}$ | **df** | **RMSEA** | **CFI** | **TLI** |
| --- | --- | --- | --- | --- | --- |
| Internalizing | 329.218 | 255 | 0.017 | 0.939 | 0.922 |
| Externalizing | 361.719 | 281 | 0.017 | 0.939 | 0.924 |
| Attention Deficit/Hyperactivity | 332.714 | 256 | 0.017 | 0.943 | 0.928 |
| Autism | 360.866 | 279 | 0.017 | 0.938 | 0.922 |
| Adjusted First-Order Factors | 518.824 | 420 | 0.015 | 0.938 | 0.922 |

Table S12. *Comparison of Effects in First-Order Factor Models, First-Order Factor Adjusted Model, and the P-Factor Model.*

|  | Mentalizing | | | | | | Social Adjustment | | | | | |
| --- | --- | --- | --- | --- | --- | --- | --- | --- | --- | --- | --- | --- |
|  | First Order | | First Order (Adj.) | | P-Factor | | First Order | | First Order (Adj.) | | P-Factor | |
|  | Est. | 95%CI | Est. | 95%CI | Est. | 95%CI | Est. | 95%CI | Est. | 95%CI | Est. | 95%CI |
| Internalizing | -.19 | -.30, -.07 | -.07 | -.19, .05 | -.07 | -.17, .03 | -.25 | -.38, -.13 | -.05 | -.18, .08 | -.08 | -.02, .17 |
| Externalizing | -.15 | -.24, -.05 | .23 | .09, .37 | .08 | -.05, .21 | -.41 | -.49, -.33 | -.14 | -.31, .03 | -.01 | -.12, .10 |
| Att. Deficit/Hyper | -.27 | -.38, -.16 | -.33 | -.52, -.14 | -.17 | -.31, -.02 | -.44 | -.53, -.35 | -.24 | -.42, -.06 | -.01 | -.12, .10 |
| Autism | -.25 | -.36, -.14 | -.15 | -.30, -.01 | -.15 | -.27, -.02 | -.38 | -.47, -.30 | -.15 | -.29, .01 | .04 | -.07, .14 |

**Note.** Est. = Completely Standardized Parameter Estimate. 95%CI = 95% Confidence Intervals. The table shows the unique association (completely standardized estimates) between (1) each separate first-order factor, mentalizing and social adjustment, (2) each first-order factor, mentalizing and social adjustment when all first-order factors are entered simultaneously, and (3) the links between each P-free factor, mentalizing and social adjustment in the P-Factor model. First Order (Adj.) = Results for the First-Order Factor Adjusted Model. Att. Deficit/Hyper = Attention Deficit/Hyperactivity Factor.

**10.0 References**

Achenbach, T. M., & Rescorla, L. A. (2013). *Manual for the ASEBA School-Age Forms and Profiles*. University of Vermont.

Brown, T. (2015). *Confirmatory Factor Analysis for Applied Research* (2nd ed.). Guilford Press.

Caspi, A., Houts, R. M., Fisher, H. L., Danese, A., & Moffitt, T. E. (2024). The General Factor of Psychopathology (p): Choosing Among Competing Models and Interpreting p. *Clinical Psychological Science*, *12*(1), 53–82. https://doi.org/10.1177/21677026221147872

Chen, F., Bollen, K. A., Paxton, P., Curran, P. J., & Kirby, J. K. (2001). Improper solutions in structural equation models. *Sociological Methods and Research*, *29*, 468–508.

Devine, R. T., & Hughes, C. (2016). Measuring theory of mind across middle childhood: Reliability and validity of the Silent Films and Strange Stories tasks. *Journal of Experimental Child Psychology*, *149*, 23–40. https://doi.org/10.1016/j.jecp.2015.07.011

Devine, R. T., Kovatchev, V., Grumley-Traynor, I., Smith, P., & Mark, L. (2023). Machine learning and deep learning systems for automated measurement of ‘advanced’ theory of mind: Reliability and validity in children and adolescents. *Psychological Assessment*, *35*(2), 165–177. https://doi.org/10.1037/pas0001186

Geiser, C. (2013). *Data analysis with Mplus*. Guilford Press.

Harden, K. P., Engelhardt, L. E., Mann, F. D., Patterson, M. W., Grotzinger, A. D., Savicki, S. L., Thibodeaux, M. L., Freis, S. M., Tackett, J. L., Church, J. A., & Tucker-Drob, E. M. (2020). Genetic Associations Between Executive Functions and a General Factor of Psychopathology. *Journal of the American Academy of Child & Adolescent Psychiatry*, *59*(6), 749–758. https://doi.org/10.1016/j.jaac.2019.05.006

Heinrich, M., Geiser, C., Zagorscak, P., Burns, G. L., Bohn, J., Becker, S. P., Eid, M., Beauchaine, T. P., & Knaevelsrud, C. (2023). On the Meaning of the “ *P-Factor*” in Symmetrical Bifactor Models of Psychopathology: Recommendations for Future Research From the Bifactor-( *S* −1) Perspective. *Assessment*, *30*(3), 487–507. https://doi.org/10.1177/10731911211060298

Muthèn, L. K., & Muthèn, B. O. (2017). *Mplus: Statistical Analysis With Latent Variables. User’s Guide.* (8th ed.). Muthèn & Muthèn.

Newsom, J. T. (2024). *Longitudinal structural equation modeling.* (2nd ed.). Routledge.

Rodriguez, A., Reise, S. P., & Haviland, M. G. (2016). Evaluating bifactor models: Calculating and interpreting statistical indices. *Psychological Methods*, *21*(2), 137–150. https://doi.org/10.1037/met0000045

Ronald, A., Happé, F., & Plomin, R. (2008). A twin study investigating the genetic and environmental aetiologies of parent, teacher and child ratings of autistic-like traits and their overlap. *European Child & Adolescent Psychiatry*, *17*(8), 473–483. https://doi.org/10.1007/s00787-008-0689-5

Woods, A. D., Davis-Kean, P., Halvorson, M., King, K., Logan, J. R., Xu, M., Bainter, S., Brown, D., Clay, J. M., Cruz, R. A., Elsherif, M. M., Gerasimova, D., Joyal-Desmarais, K., Moreau, D., Nissen, J., Schmidt, K., Uzdavines, A., Van Dusen, B., & Vasilev, M. (2021). Missing data and multiple imputation decision tree. *PsyArXiv*. https://doi.org/10.31234/osf.io/mdw5r
